# Endovascular management of chronic internal carotid occlusion with Penumbra system

**Published:** 2017-01-05

**Authors:** Masoud Mehrpour

**Affiliations:** ^1^ Department of Neurology, School of Medicine, Iran University of Medical Sciences, Tehran, Iran

**Keywords:** Carotid Occlusion, Revascularization, Penumbra System

Carotid occlusion is a common disease and its incidence is about 6 in 100000. Annual risk of stroke is between 3% and 10% in older than 60 years with carotid occlusion and recurrent ischemic event have been reported in 25% of symptomatic carotid occlusion.^1^ Since many years ago it has been advocated in complete arterial occlusion, there was no chance for emboli to pass into the distal circulation. Therefore, a complete occlusion of the internal carotid artery (ICA) has been regarded as no need to follow-up and the natural history and optimal management of this condition remain undetermined up to now.^2^

Nowadays, chronic occluded vessels could be revascularized by endovascular techniques. It has been successfully used in peripheral and coronary arteries.^3^ The recent development of endovascular therapy has enabled the recanalization of carotid occlusion technically although it needs more study to evaluate its efficacy.

In this case presentation, we describe new technique in opening symptomatic chronic carotid occlusion with the aim of reducing risk of distal embolization using the Penumbra thromboaspiration system (Alameda, California, USA).

A 56-year-old man was refereed with history of repeated stroke in the territory of left anterior circulation since 1 month ago. He was treated medically and evaluated for risk factors of stroke. He was heavy smoker. Carotid duplex study showed bilateral carotid occlusion. Digital subtraction angiography confirmed bilateral ICA occlusion from the origin. ICAs were filled in cavernous part by retrograde flow from ophthalmic arteries of external carotid arteries (ECAs). The patient had repeated transient ischemic attack in spite of best medical treatment. Due to the deterioration of the patient, it was decided to treat the patient by endovascular revascularization. The risks and benefits of the procedure were explained to the patient and his relatives. An informed consent was obtained from the patient.

The patient underwent endovascular revascularization, he was under aspirin and plavix for 2 weeks. The procedure was performed under general anesthesia and via the percutaneous transfemoral route. Heparin was injected intravenously to maintain activated clotting time between 200 and 250 seconds. Guiding catheter 8 Fr was placed in the left common carotid artery (CCA). With contrast injection it was shown that there was left ICA occlusion at origin (Figure 1-A). For reducing the risk of dissection because it is impossible to show the roadmap for determining the right way to pass the true lumen (Figure 1-B). With the coaxial system 3 Max reperfusion catheter (Alameda, California, USA) was placed near to the occlusion and microwire 0.014 pilot was introduced to the catheter to approach to the occlusion part. Microwire probing the occlusion part to find a passing way. During navigating and probing the occlusion the Penumbra system was working for thromboaspiration continuously. It was applied through the Penumbra system. After passing the microwire from occlusion part (Figure 1-C), the 3 Max catheter was enhance over the microwire in association with negative pressure through catheter by Penumbra system (Figure 1-D). Navigation of catheter with suction decreased the risk of distal embolization. With passing the catheter to distal part of ICA, it was evaluated the patency of distal ICA (Figure 1-E). It was confirmed that catheter is in true lumen by control angiography (Figure 1-F). Fortunately, the rest of the lumen was opened.

3 Max catheters was retrieved under suction of Penumbra system (Figure 1-G). Then WALLSTENT 7 mm × 40 mm was deployed in left ICA to CCA (Figure 1-H). Then balloon post dilatation 5 × 20 was performed. ICA occlusion was repaired completely (Figure 1-I). Carotid occlusion revascularization was successful.

The patient condition was good and stable after recovery. There was no new neurological deficit, and he was referred to neurologic intensive care unit. 

Brain magnetic resonance imaging (MRI) showed no new lesion and carotid duplex and magnetic resonance angiographic showed patency of left ICA. The patient got better during hospitalization. He could walk independently after 1 week and he was discharged in good condition. 

It seems still revascularization of carotid occlusion is a taboo. Unfortunately, the natural history of patients with carotid artery occlusion is poorly understood, so there are continuing areas of debate in decision to recanalization of the occluded ICA. It is preferred to treat them medically as the difficulty and risks of surgery are thought to outweigh the natural history of the condition. Why carotid occlusion is abandoned to treat. It may be due to previous study which could not prove the efficacy of ECA/ICA bypass surgery.^4^

There is growing case reports and case series of carotid occlusion revascularization with intervention in recent years. They showed carotid occlusion revascularization is feasible and safe.^5^^-^^7^

There is always risk of dissection and distal emboli. Kao, et al.^7^ advocated that excessive rotational or drilling motion of the wire was avoided, but successive small penetrate-and-advance steps carefully along the imaginary tract of the occluded vessel segment can pass the occlusion. Namba, et al.^3^ suggest that initial penetration of the occluded stump from the anterior side will provide maximal chance to access the “true lumen.” Rostambeigi, et al.^8^ used ultrasonography to navigate and pass the carotid occlusion.

The major concern in recanalizing chronic ICA occlusion is the possibility of causing distal embolism from the stump. Terada, et al.^9^ believe that embolic complication appear during angiography just after angioplasty for the occluded point from the guiding catheter placed in the CCA. Forceful injection of the contrast agent might cause the emboli remaining in the stump of the recanalized lumen to migrate. Although Spearman, et al.,^10^ believed in chronic ICA occlusion, the thrombotic content in the stump is further organized, and the possibility of releasing embolic debris during device manipulation should be minimal. In addition, the antegrade flow after guide wire crossing and gentle undersized predilatation is usually sluggish, with low risk of carrying emboli downstream.

For decreasing the risk of distal emboli there was two strategies that was applied by different authors. Distal protection and proximal protection is recommended.^6^ In this case, the different strategy was applied by Penumbra system to decrease the risk of distal emboli. Using the Penumbra system, it seems to reduce the risk of distal emboli and facilitate navigation and passing through the occlusion. Penumbra system is safe and effective in patients experiencing acute ischemic stroke secondary to large vessel occlusive disease, it also has been used for other occlusive disease like sinus thrombosis.^11^ Penumbra system can be used to revascularization of chronic carotid occlusion with low risk of distal emboli. Although, this is a preliminary report but it seems this technique is safe and feasible.

**Figure 1:**
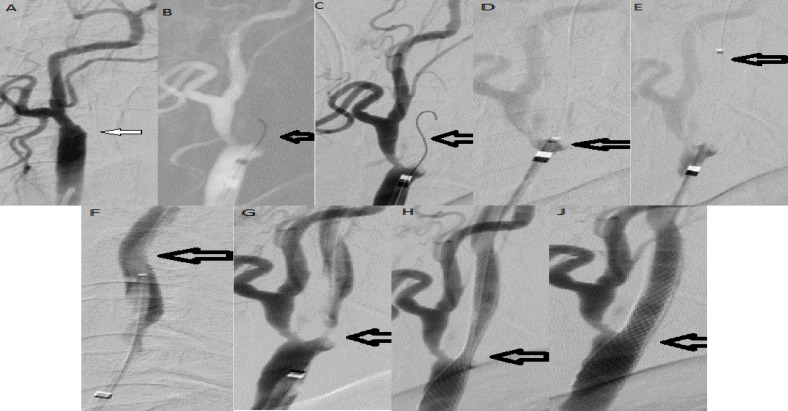
(A) Left internal carotid artery (ICA) complete occlusion (arrow), (B) passing microwire through the occlusion, (C) control digital subtraction angiography after microwire position, (D) approaching 3 Max catheter with Penumbra system, (E) passing through the occlusion, (F) control catheter position in ICA, (G) retrieve 3 Max, (H) deploying the stent, (I) post ballon angioplasty final result

## Notes:

**How to cite this article:** Mehrpour M. Endovascular management of chronic internal carotid occlusion with Penumbra system. Iran J Neurol 2017; 16(1): 50-2.
